# Correction: Antibody response, associated symptoms and profile of patients presumably infected by SARS-CoV-2 with taste or smell disorders in the SAPRIS multicohort study

**DOI:** 10.1186/s12879-023-08450-2

**Published:** 2023-07-26

**Authors:** Julien Ramillon, Xavier de Lamballerie, Olivier Robineau, Hélène Blanché, Gianluca Severi, Mathilde Touvier, Marie Zins, Fabrice Carrat, the SAPRIS-SERO study group, Fabrice Carrat, Gianluca Severi, Mathilde Touvier, Marie Zins, Hélène Blanché, Xavier de Lamballerie, Alexandra Rouquette, the SAPRIS study group, Fabrice Carrat, Gianluca Severi, Mathilde Touvier, Marie Zins, Mireille Pellicer, Julien Allegre, Mélanie Deschasaux, Delphine Rahib, Nathalie Lydie, Olivier Robineau, Liza Belhadji, Laetitia Ninove, Nathanaël Lapidus

**Affiliations:** 1 Département de Santé Publique, Hôpital Saint-Antoine, AP-HP.Sorbonne Université, 75012 Paris, France; 2 Unité Des Virus Emergents, UVE: Aix Marseille Univ, IRD 190, INSERM 1207, IHU Méditerranée Infection, 13005 Marseille, France; 3 Sorbonne Université, Inserm, Institut Pierre Louis d’Epidémiologie Et de Santé Publique IPLESP, AP-HP.Sorbonne Université, 75012 Paris, France; 4 EA2694, Univ Lille, Centre Hospitalier de Tourcoing, Tourcoing, France; 5 Fondation Jean Dausset-CEPH (Centre d’Etude du Polymorphisme Humain), CEPH-Biobank, Paris, France; 6 CESP UMR1018, Université Paris-Saclay, UVSQ, Inserm, Gustave Roussy, Villejuif, France; 7 Department of Statistics, Computer Science and Applications, University of Florence, Florence, Italy; 8 Sorbonne Paris Nord University, Inserm U1153, Inrae U1125, Cam, Nutritional Epidemiology Research Team (EREN), Epidemiology and Statistics Research Center - University of Paris (CRESS), Bobigny, France; 9 Population-Based Epidemiological Cohorts, UMS 11, Paris-Saclay University, Versailles St Quentin University, Université de Paris, Inserm, Villejuif, France

**Correction: BMC Infect Dis 23, 228 (2023)**

**https://doi.org/10.1186/s12879-023-08162-7**

The original publication of this article [1] contained an incorrect acknowledgement section as several collaborators were omitted.

The missing collaborators are shown in the additional file. The original article has been updated.

## Supplementary Information

**Additional file 1.**
