# Supplementary material for: Antibody response, associated symptoms and profile of patients presumably infected by SARS-CoV-2 with taste or smell disorders in the SAPRIS multicohort study
Source: BMC Infect Dis. 2023 Apr 14;23:228. doi: 10.1186/s12879-023-08162-7 (PMC10103027; doi:10.1186/s12879-023-08162-7)
Supplement: Supplementary file 1 — Additional file 1: Supplementary Figure S1A. Symptoms associations in participants with positive ELISA-S and taste or smell disorders. Supplementary Figure S1B. Symptoms associations in participants with positive ELISA-S and no taste or smell disorders. Table S1. Characteristics of participants with and without taste or smell disorders (TSD) in the sensitivity analysis conducted with multiple imputation to handle missing values. Categorial variables are reported as average counts (percentages of non-missing values) and quantitative variables as median [Q1–Q3]. Table S2. Distribution of serological titers and associations with taste or smell disorders (TSD) adjusted for sex, age, smoking status, alcohol consumption and BMI in the sensitivity analysis conducted with multiple imputation to handle missing values. [file 12879_2023_8162_MOESM1_ESM.docx]

**Supplementary Figure S1A.** Symptoms associations in participants with positive ELISA-S and taste or smell disorders


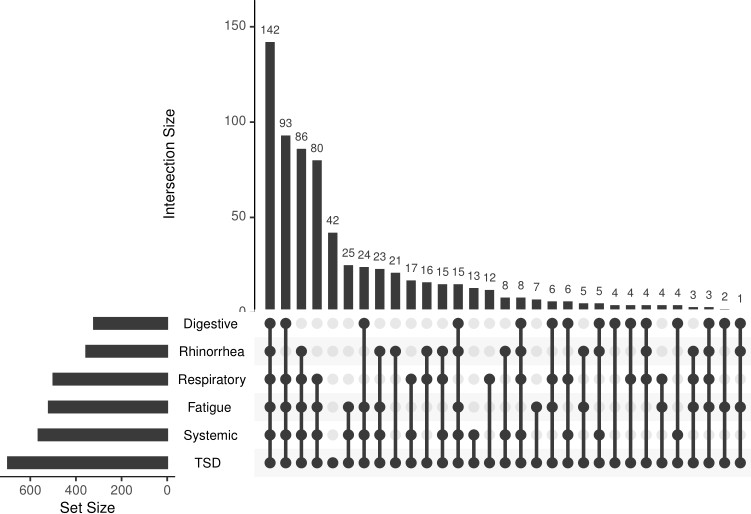


**Supplementary Figure S1B.** Symptoms associations in participants with positive ELISA-S and no taste or smell disorders


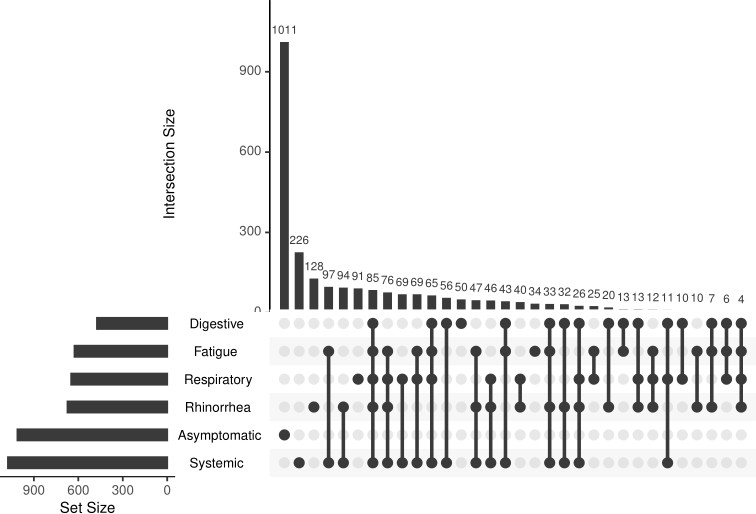


**Table S1.** Characteristics of participants with and without taste or smell disorders (TSD) in the sensitivity analysis conducted with multiple imputation to handle missing values. Categorial variables are reported as average counts (percentages of non-missing values) and quantitative variables as median [Q1 – Q3]

|  | Characteristics of patients  (30 iterations with 3,660 participants) | | Multivariable analysis with imputation data | |
| --- | --- | --- | --- | --- |
|  | With TSD  (n=22,916) | Without TSD  (n=86,884) | OR | 95% CI |
| Sex |  |  |  |  |
| Men | 6,623 (28.9%) | 27,127 (31,2%) | 1 |  |
| Women | 16,293 (71.1%) | 59,757 (68.8%) | 1.24 | 1.03 - 1.49 |
| Smoking status |  |  |  |  |
| Non smoker or former smoker | 20,089 (87.7%) | 79,374 (91.4%) | 1 |  |
| Active smoker | 2,827 (12.3%) | 7,510 (8.6%) | 1.59 | 1.22 - 2.10 |
| Alcoholic consumption |  |  |  |  |
| 2 or less drinks a day | 18,833 (82.2%) | 75,060 (86.4%) | 1 |  |
| 3 or more drinks a day | 4,083 (17.8%) | 11,824 (13.6%) | 1.37 | 1.09 - 1.74 |
| BMI |  |  |  |  |
| Underweighted or normal  (BMI < 25) | 14,720 (64.2%) | 56664 (65.2%) | 1 |  |
| Overweighted or obese (BMI ≥ 25) | 8,196 (35.8%) | 30220 (34.8%) | 0.98 | 0.82 - 1.16 |
| Age |  |  |  |  |
| 30 years old |  |  | 1.85 | 1.36 - 2.53 |
| 40 years old |  |  | 1 |  |
| 50 years old |  |  | 1.84 | 1.58 - 2.13 |
| 60 years old |  |  | 3.87 | 3.21 - 4.67 |
| 70  years old |  |  | 1.70 | 1.39 - 2.08 |

**Table S2.** Distribution of serological titers and associations with taste or smell disorders (TSD) adjusted for sex, age, smoking status, alcohol consumption and BMI in the sensitivity analysis conducted with multiple imputation to handle missing values.

|  | Without TSD | | With TSD | | Multivariable analysis | |
| --- | --- | --- | --- | --- | --- | --- |
|  | Median | Q1-Q3 | Median | Q1-Q3 | OR | 95% CI |
| ELISA-S | 1.90 | 1.41 - 3.02 | 2.98 | 1.88 - 4.82 | 1.31 | 1.26 - 1.36 |
| ELISA-NP | 0.61 | 0.38 - 1.40 | 2.19 | 1.28 - 3.79 | 1.37 | 1.33 - 1.42 |
| SN | 10 | 10 - 20 | 40 | 20 - 160 | 1.34 | 1.29 - 1.39 |
