# Supplementary material for: Correction: Antibody response, associated symptoms and profile of patients presumably infected by SARS-CoV-2 with taste or smell disorders in the SAPRIS multicohort study
Source: BMC Infect Dis. 2023 Jul 26;23:492. doi: 10.1186/s12879-023-08450-2 (PMC10373370; doi:10.1186/s12879-023-08450-2)
Supplement: Supplementary file 1 — Additional file 1. [file 12879_2023_8450_MOESM1_ESM.docx]

**Missing collaborators in the original publication**

**SAPRIS study group**

Alexandra Rouquette

**SAPRIS-SERO study group**

Mireille Pellicer, Julien Allegre, Mélanie Deschasaux, Delphine Rahib, Nathalie Lydie, Olivier Robineau, Liza Belhadji, Laetitia Ninove
